# Perspectives of policy and political decision makers on access to formal dementia care: expert interviews in eight European countries

**DOI:** 10.1186/s12913-017-2456-0

**Published:** 2017-08-03

**Authors:** Anja Broda, Anja Bieber, Gabriele Meyer, Louise Hopper, Rachael Joyce, Kate Irving, Orazio Zanetti, Elisa Portolani, Liselot Kerpershoek, Frans Verhey, Marjolein de Vugt, Claire Wolfs, Siren Eriksen, Janne Røsvik, Maria J. Marques, Manuel Gonçalves-Pereira, Britt-Marie Sjölund, Bob Woods, Hannah Jelley, Martin Orrell, Astrid Stephan, the ActifCare Consortium

**Affiliations:** 1 Martin Luther University Halle-Wittenberg, Institute of Health and Nursing Sciences, Magdeburger Straße 8, D-06112 Halle (Saale), Germany; 2 Dublin City University, School of Nursing and Human Sciences, Dublin, Ireland; 3 IRCCS S. Giovanni di Dio “Fatebenefratelli”, Brescia, Italy; 4 Alzheimer Center Limburg, Maastricht University, Maastricht, The Netherlands; 5 Norwegian National Advisory Unit on Ageing and Health, Vestfold Hospital Trust, Oslo, Norway; 6 Department of Geriatric Medicine, Oslo University Hospital, Oslo, Norway; 7 CEDOC, Chronic Diseases Research Center, Nova Medical School/Faculdade de Ciências Médicas, Universidade Nova de Lisboa, Lisbon, Portugal; 8 Aging Research Center (ARC), Department of Neurobiology, Care Sciences and Society (NVS), Karolinska Institutet and Stockholm University, Stockholm, Sweden; 9 Faculty of Health and Occupational Studies, Department of Health and Caring Sciences, University of Gävle, Gävle, Sweden; 10 Bangor University, Dementia Services Development Centre, Bangor, UK; 11 Nottingham University, Institute of Mental Health, Nottingham, UK

**Keywords:** Dementia, Access to formal dementia care, Expert interviews

## Abstract

**Background:**

As part of the ActifCare (ACcess to Timely Formal Care) project, we conducted expert interviews in eight European countries with policy and political decision makers, or representatives of relevant institutions, to determine their perspectives on access to formal care for people with dementia and their carers.

**Methods:**

Each ActifCare country (Germany, Ireland, Italy, The Netherlands, Norway, Portugal, Sweden, United Kingdom) conducted semi-structured interviews with 4–7 experts (total *N* = 38). The interview guide addressed the topics “Complexity and Continuity of Care”, “Formal Services”, and “Public Awareness”. Country-specific analysis of interview transcripts used an inductive qualitative content analysis. Cross-national synthesis focused on similarities in themes across the ActifCare countries.

**Results:**

The analysis revealed ten common themes and two additional sub-themes across countries. Among others, the experts highlighted the need for a coordinating role and the necessity of information to address issues of complexity and continuity of care, demanded person-centred, tailored, and multidisciplinary formal services, and referred to education, mass media and campaigns as means to raise public awareness.

**Conclusions:**

Policy and political decision makers appear well acquainted with current discussions among both researchers and practitioners of possible approaches to improve access to dementia care. Experts described pragmatic, realistic strategies to influence dementia care. Suggested innovations concerned how to achieve improved dementia care, rather than transforming the nature of the services provided. Knowledge gained in these expert interviews may be useful to national decision makers when they consider reshaping the organisation of dementia care, and may thus help to develop best-practice strategies and recommendations.

**Electronic supplementary material:**

The online version of this article (doi:10.1186/s12913-017-2456-0) contains supplementary material, which is available to authorized users.

## Background

For people with dementia, home care may be regarded the desired way of caring, especially for people in the early and middle stages of the disease. Home care is beneficial because many people with dementia prefer to live at home for as long as possible cared for by their family, it may provide better quality of life, and it may be less expensive than institutional care [1]. Informal carers such as family members often provide home care. These informal carers are often partners of advanced age who face health and social care challenges themselves, or are adult children with multiple responsibilities regarding work, family, finances etc. Informal carers can experience high levels of stress, depression, social isolation and physical health problems [2].

To realize home care, appropriate support services for both people with dementia and their carers are necessary. Many countries have acknowledged this and have adopted policies to develop better services and reduce institutionalisation [3]. However, research has revealed that people with dementia and their carers are not receiving services of the type and quality that they need, and that they experience much difficulty accessing home- and community-based services [4]. Thus, access to formal dementia care remains a crucial issue in fulfilling care needs, increasing the quality of life for people with dementia, and reducing the burden on resources for example by preventing nursing home placement.

In health and social care systems, policy and political decision makers are usually in a core position to prepare, influence and make decisions on dementia care. Such experts possess valuable specific knowledge and experiences on how to structure and shape dementia care. Therefore, in the current study, we were interested in the perspectives of policy and political decision makers regarding access to formal dementia care in their country, and conducted expert interviews in eight European countries. Specifically we were interested in innovative ideas, strategies or suggestions of experts regarding complexity and continuity of care, formal services, and public awareness.

## Methods

As part of ActifCare (ACcess to Timely Formal Care) [5], an EU Joint programme on neurodegenerative disease research (JPND)-funded project, we conducted qualitative semi-structured interviews in eight European countries (Germany = DE, Ireland = IE, Italy = IT, Netherlands = NL, Norway = NO, Portugal = PT, Sweden = SE, United Kingdom = UK) with expert policy and political decision makers to determine their perspectives on access to home- and community-based formal care for people with dementia and their carers. ActifCare specifically focuses on people with dementia living at home who do not as yet access formal care services but may do so in the near future. The ActifCare concept of formal care refers to home care, day care services, in-home long-term medical nursing, social care structures and processes, and excludes domestic home help, housekeepers, volunteers, support groups, transport services, and meal programmes [5]. Thus, formal care includes help and services that are provided by health or social care professionals on account of the person’s dementia.

Expert interview is a common method in health services and public health research, including studies on care for people with dementia [6] and on the views of healthcare policy and decision makers [7, 8]. The interviews concerned the experts’ specific knowledge and experiences which result from the actions, responsibilities, or obligations of the experts’ functional status within a dementia care organisation or institution. They did not concern the experts themselves, not the individual or single case, but the expert as a source of information [9].

### Sample

The research group in each country was expected to interview three to five carefully selected experts. A total of 38 expert interviews were conducted between September 2015 and January 2016. Characteristics of the experts are displayed in Table 1.

**Table 1:** Characteristics of the experts

	Total	DE	IE	IT	NL	NO	PT	SE	UK
Experts (n)	38	6	7	4	4	5	4	4	4
Gender									
Female	25	4	5	2	2	5	2	3	2
Male	13	2	2	2	2		2	1	2
Age (years)									
< 40	2		1		1				
40–44	2	1			1				1
45–49	2		1						
50–54	8	3	1			2	2		
55–59	11	1	3			2	1	1	3
60–64	7	1	1	2	2	1			
> =65	6			2	-		1	3	
Years of professional experience, range	12–63	15–38	12–35	26–40	17–38	25–39	29–40	30–63	21–34
Years of experience with dementia care, range	0.5–38	3–23	1–30	17–30	14–38	0.5–25	11–30	30–37	5–33
Years of experience in current position, range	0.5–35	1–23	0.5–10	6–22	1–12	0.5–7	5–15	15–35	2–19
Type of experts^a^ (n)									
Policy maker	7		2	1			1		3
Political decision maker (elected)	2					1		1	
Political decision maker (not elected)	7	3			1	3			
Advisory board	6		1	4	1				
Academic researcher	1							1	
Representative of NGO/Alzheimer society	11	1	2	1	1	1	3	1	1
Representative of insurance company	3	2			1				
Representative of dementia care provider	2				1		1		
Representative of funding organization	2		2						
Representative of umbrella organization providing advice and collecting data on dementia care	1							1	
Experts (n) operating on …									
national level	30	4	7	3	3	2	3	4	4
regional level	4	1			1	1	1		
local level	4	1		1		2			
Mode of interview									
face-to-face	17		5	3	1	4	4		
telephone	21	6	2	1	3	1		4	4
Duration of interview in min, range, mean	20–110, 53.5	20–50, 30.8	45–55, 52.1	95–110, 102.5	24–50, 40.3	50–90, 65.0	48–70, 59.5	45–65, 57.5	20–45, 32.5

Note: *DE* Germany, *IE* Ireland, *IT* Italy, *NL* Netherlands, *NO* Norway, *PT* Portugal, *SE* Sweden, *UK* United Kingdom, *NGO* Non-governmental organisation

^a^Multiple responses were possible

Selection of the experts was at the discretion of the individual countries, however, the following guides on determining relevant experts were provided: (1) Being in a unique position to influence national policies and decision making about dementia care; (2) possessing special knowledge which is not accessible to everybody; [10] (3) possessing an institutionalized authority to be influential in a relevant way, i.e., have decisional power [9]. Therefore, participants could include direct policy makers (elected) and representatives of ministries or governmental departments in permanent positions (non-elected) as well as e.g. representatives of relevant NGO’s, Alzheimer societies or umbrella organizations providing formal dementia care. Experts could be identified at a national, regional, or local level, depending on the specific structure of the dementia care system in the ActifCare countries, and recruited from both the level of immediate decision-making and of preparing decisions [9]. Informed consent of the experts was obtained regarding audio recording and transcription. The experts were assured that all data protection guidelines are met, and that quotations could be checked and proof-read by the expert before publication. When presenting the findings, experts are identified by their country code and a consecutive number referring to a particular participant.

Interviewers were members of the research group in each country, including research nurses, psychologists, sociologists or physicians by training, who were well acquainted with conducting semi-structured guided interviews.

### Interview guide

An interview guide (see Additional file 1) was developed by the ActifCare research group of Germany in close collaboration with all partners.

The interview topics were built on insights from previous ActifCare focus group interviews with people with dementia, informal carers, and health and social care professionals [11]. The analysis of the focus group data revealed important barriers and facilitators across ActifCare countries that may be specifically addressed by strategic policy measures on a system level. These topics, “Complexity and Continuity of Care”, “Formal Services” and “Public Awareness”, formed the basis for the expert interviews. Experts were asked to use their professional experience to provide innovative ideas, strategies and suggestions to influence each.

Initial drafts of the interview guide were discussed with two German policy or political decision makers. These persons were acquainted with both academic and structural knowledge of the dementia field. They did not subsequently take part in the interviews. The aim of this methodological step was to review the interview topics from an external perspective and to ask for suggestions of additional topics that should be addressed. Once finalized, the interview guide was provided in English, and was subsequently translated by the partners into their national languages.

As in any guided interview, the interview guide with its questions and phrases could be adapted to ensure relevance for the specific expert and the specific situation.

### Procedure

Researchers in each country identified and approached the experts and conducted the interviews in their country. Initial contact with possible experts was made by e-mail, telephone, or personal visits. Interviews were conducted face-to-face or by telephone on a scheduled date at the convenience of the expert. At the discretion of the interviewer, or at request of the expert, the general content of the interview, i.e., the three topics, was sent to the expert in advance. Throughout the interview, it was repeatedly made clear to the expert that the interest of the interview was on formal services provided for people with dementia living at home. All interviews were audio recorded, and subsequently intelligent verbatim transcripts were prepared.

### Analysis

A stepwise analytical procedure was applied. First, researchers in each country analysed their own interview transcripts using inductive qualitative content analysis, which involves using open coding and deriving themes and categories directly from the material [12]. The findings of each country were reported in a narrative and comprehensive way. Themes were described in terms of their content, meaning, reach (and relation to other themes, if applicable), and appropriate anchor examples were selected from the transcripts to illustrate each theme. The findings were translated into English by each country.

Second, the research group of Germany as the leader of the expert interview ActifCare work package generated a cross-national synthesis based on the translated country-specific findings, focusing on similarities in themes across countries. This synthesis was evaluated and discussed by all partners to ensure that final interpretations reflected the meaning of each country’s results. Evaluation and discussion first took place in a face-to-face workshop during an ActifCare project meeting and subsequently through structured e-mail conversations where the written synthesis was reviewed and amended until consensus was reached.

When presenting the results, themes that were relevant in more than one country are identified. To illustrate these themes, either direct expert quotations serving as anchor examples or listings of expert statements for the theme are provided.

## Results

Table 2 provides a summary of the ten common themes and two additional sub-themes identified across countries, along with either direct expert quotations or listings of expert statements. Further description and explanation for each theme follow:

**Table 2:**
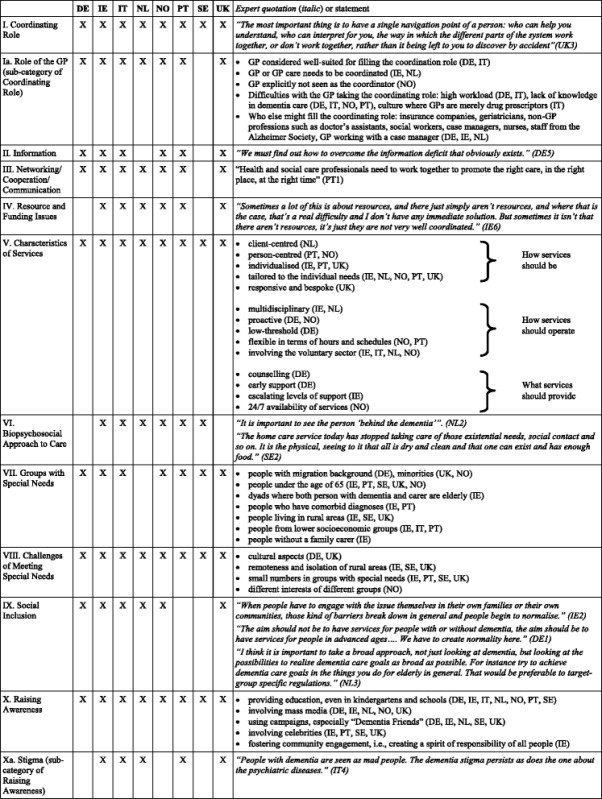
Identified themes and corresponding expert quotations or statements by country

Note: Direct expert quotations are identified by country code (DE Germany, IE Ireland, IT Italy, NL Netherlands, NO Norway, PT Portugal, SE Sweden, UK United Kingdom) and a consecutive number. See Results for additional direct expert quotations

### I. Coordinating Role

One overriding theme was the need for a coordinating role, i.e., someone to help people with dementia and their carers navigate health and social care systems. Experts highlighted the need for a person or an institution that can guide through available supports and services, and can coordinate and facilitate access.

*“I’m very taken with the notion of some kind of link person who links people with dementia into the system and navigates it for them.” (IE1).*

*“Experts must help them to understand what’s best for them in that moment” (IT1).*

*“Carers and also persons with dementia experience that it is not so easy to navigate because there is a lack of contact persons…” (NO3).*

*“…that is why I think there should be a 'way in', someone who is like at the heart of things, a coordinator that sees the whole picture and knows where to get help.” (SE3).*

The specifics as to what structures or systems are needed to realise such a coordinating role differed between countries. IE experts referred to a Dementia Key Worker role. This was a signposting only role according to some experts, while others felt that it should include case management. UK experts advocated for a Dementia Advisor, NO experts for a designated Dementia Coordinator and/or a Dementia Team, and PT experts for a Dementia Advisor and/or multidisciplinary teams (general practitioners [GPs], social workers, nurses, etc.) trained specifically in dementia within primary care or community services.

*“One aspiration is a Dementia Advisor… Giving people a 50 page pamphlet on dementia when they’ve asked a specific question isn’t appropriate… Having a single person, so a person could get advice would be helpful.” (UK2).*

*“In my opinion, primary care services should have multidisciplinary teams in each health centre trained specifically on dementia to whom people with this condition can be referred to. A kind of a “leader” or “dementia advisor” within primary care or community services” (PT2).*

SE experts also favoured an approach with teams consisting of a qualified nurse with training in dementia and a health and social care assessor to attend to care needs.

*“Something I believe in is a home care dementia team.” (SE4).*

IE experts further debated whether the role should have a clinical background, whether the role should lie inside or outside the health and social care system, and whether multiple roles are in fact being alluded to when describing what the coordinating role is supposed to cover, e.g. a Dementia Advisor for information, education, post-diagnostic support and a Case Manager for more complex health and social care support once formal systems have been engaged. NL experts indicated that it is important that someone takes the coordinating role, but that it is not important who this is (case manager, geriatrician, GP etc.)

### Ia. Role of the GP.

Role of the GP emerged as an important sub-theme in relation to “Coordinating Role”. In DE and IT, experts preferred to see the GP taking over the coordinating role.

*“In my opinion, GPs play a different, very significant role. GPs should be brought in because often they can realize access.” (DE6).*

*“It is necessary to have a person who is a constant and continue referral point (…) this person cannot be any other than the GP (…) he should not delegate” (IT4).*

At the same time, experts in several countries also acknowledged difficulties with the GP taking over the coordinating role (Table 2). As a consequence, experts elaborated on who else might take over the coordinating role.

*“The GP, for example, or the community nurse, or whoever has a person they can go to who will know all the major services in the area and all that kind of stuff, and I don’t mean somebody who has four other jobs and is given this job.” (IE4).*

*“And which profession that is or which institution, in my opinion that is secondary. (…) And for one person, it can be someone from the GPs office, and that does not have to be the GP itself, it can be the doctor’s assistant. Or it can be a social worker, or someone from the civil society, the Alzheimer Society.” (DE6).*

In IE and NL, the GP was considered to be one of the professionals who needed to be coordinated by e.g. case managers, dementia teams or networks. In NO the GP was explicitly not seen as the coordinator, because most of the NO municipalities have dementia teams or dementia coordinators.

### II. Information

Experts pointed out that information about available services and about benefits of services are crucial when dealing with complexity and continuity of care. Access to information was seen as a precursor to service access itself. The theme includes expert reflections of the need to create transparency by collecting and publishing information in an adequate manner.

*“Information about the nature of the condition, the trajectory of the condition, about what services and supports are available, but also about the potential benefits of those services and supports, and at the time that those supports may optimise the benefit for the person. So just telling somebody that a support or service is there isn't enough. You really need to explain the benefit of it, and when it might be the best time to engage in it.” (IE4).*

One idea suggested by experts from PT and NO was the creation of online platforms or websites that contain information regarding existing services and supports. Problems were simultaneously acknowledged, for example, many people with dementia or their carers may not have access to computer, or may not be able to search for information themselves, or may not know what information is needed.

*“It would be important to set up an online platform that contains the various existing services. Of course there are many people who still do not have access to computer but there is always a family member, a technician or an association that can provide the information” (PT3)*

### III. Networking/Cooperation/Communication

Experts referred to the necessity of networking, cooperation and communication among diverse professions and different sectors involved in care. Although the national structures of dementia care differ between countries and thus the specific professions, sectors or structural (sub-)systems named by the experts also differed between countries, a major similarity was that the experts agreed that those different professions, sectors or structural (sub-)systems should be required to work together. For example, experts from IE named the public and private services within the formal healthcare system, along with the voluntary and community sector, experts from PT named primary, specialized (secondary) care and community health and social services, experts from NO named municipalities, and experts from DE named inpatient and outpatient care and specific medical and therapeutic professions.

*“We should have networks where counsellors of care insurances, case managers, care centres providing inpatient and outpatient care, including day care, night care, voluntary neighbourly help, and so on, work hand in hand.” (DE2).*

*“You need systems where they are talking to each other …” (IE4).*

*“Fragmentation is inevitable, organization-wise. There are simply different disciplines needed at different stages of time. Sometimes multidisciplinary collaboration is required, and you must organise this.” (NL1).*

*“We need to coordinate, for instance, primary care with secondary (specialized) care and social care... There are places where the coordination is working reasonably well, but there are other places where it simply does not exist…”(PT1).*

### IV. Resource and Funding Issues

Experts identified a lack of and limitations with funds and resources as important reasons for not adequately dealing with complexity and continuity of dementia care. They expressed the view that dementia may not be a high priority area for health care systems or at a governmental level, or that dementia is competing for resources with other chronic conditions.

*“I think one of the difficulties is that obviously there are resource implications for this and it’s where these resource implications end up… I mean, that’s usually the stumbling block.” (IE5).*

*“You cannot start to create new things without examining the existent resources. You cannot ‘make an omelette without eggs’.” (PT1).*

It was also suggested that reallocation and reorganisation of funds and resources may increase efficiency. For example, IE experts mentioned using homecare package funding in a different way, or spending resources earlier, in home care, rather than later, in long-term care and hospital-based services. IT experts demanded re-visiting the way the system distributes the economic benefit check (*assegno di accompagnamento*, a monetary compensation for invalidity issued in a rather bureaucratic process), and UK experts advocated for the integration of social and healthcare budgets and the creation of personal budgets as a means of ensuring the available funds are spent most appropriately. In contrast, one expert from NO perceived dementia services as having received increased funding. It was claimed that this was due to changes in attitudes towards dementia that have led to the disease becoming a higher priority in this country.

### V. Characteristics of Services

Experts reflected on characteristics that services should have, and a list was compiled of the actual terms used by the experts to describe the desired characteristics (Table 2). A first group of terms referred to the opinion of the experts how services should be, described as e.g. client- or person-centred or tailored to individual needs. A second group provided keywords regarding how services should operate, e.g. multidisciplinary, or involving the voluntary sector. A third group detailed specific examples of what services should provide. Here, DE experts highlighted the necessity of counselling and early support….

*“Counselling is of course an important factor to explain complex issues and to support people here.” (DE3).*

*“We have the idea… to enter earlier and provide help earlier. We want to strengthening abilities by providing early support.” (DE5).*

…IE experts spoke about the need for escalating levels of care and how best to monitor and respond to that need….

*“You could imagine at the start of their journey people might be more self-directing and be able to, I suppose, navigate the system with their family more easily, whereas as the dementia progresses they might need more help in navigating the system and that there would be a case management system that could start quite light-touch and as they kind of progress through, that it could become more intensive until you kind of get to the palliative care end.”(IE3).*

…and NO experts were concerned with the availability of services at daytime and on evenings, on weekdays and on weekends alike.

### VI. Biopsychosocial Approach

Experts advocated for a biopsychosocial approach to care. In addition to medical care needs, social, emotional and psychological needs must also be taken into account when creating services. In this regard, experts in PT and NO stressed the need to promote continuous education and training in geriatric issues and in dementia for those who provide services or care.

*“The home care service today has stopped taking care of those existential needs, social contact and so on. It is the physical, seeing to it that all is dry and clean and that one can exist and has enough food.” (SE2).*

*“The training should include the development of core competencies, including communication skills and competencies to provide person-centred care and a broader perspective about people with dementia instead of a strictly biomedical approach.” (PT2)*

### VII. Groups with Special Needs

Experts identified groups with special needs beyond the core group of interest. IE experts named many different groups, while DE and IT experts named only one group and NL experts named none. Some of the different groups from the IE experts were also mentioned in other countries. The group most often mentioned was younger people with dementia, or people with an earlier onset of dementia under the age of 65, which was mentioned by experts in five countries. Experts in three countries referred to people with migration background or minorities, people living in rural areas, and people from lower socioeconomic groups, respectively (Table 2). While there was some agreement in kind of special groups, there was no agreement among the experts of different countries as to whether special services should be created for any or all special groups. In DE, the experts‘perspective was that integrated and inclusive services should be created. In PT, experts said that special services are needed and that certain groups (e.g. people with early onset dementia) should be considered separately due to their unique characteristics. In IE, experts felt that implementing a truly needs-focused, tailored, coordinated and responsive dementia care system would address many of the ways in which these groups are currently disadvantaged. In UK and NO, the experts cautioned against making too many sub-categories, and stressed the need for person-centred care rather than making assumptions based on broad categories. In SE, the experts had doubts that in an adult day care centre special activities for special groups were necessary. It was considered more important to take into account the stage of dementia or the type of dementia. SE experts recommended creativity to find unique solutions when designing activities for diverse people.

*“What we have to do is have good quality services for everybody, but adjust those services for individuals” (UK1).*

*“No, one should not combine, but sometimes they are (dementia patients) so few that it is difficult to have completely separate activities, but then one should have them organised in the day care centre so that the people with dementia are in a group within the larger group.” (SE3)*

### VIII. Challenges of Meeting Special Needs

Experts alluded to challenges of meeting special needs. For example, DE and UK experts referred to potential cultural barriers associated with people with a migration background. IE, SE and UK experts referred to remoteness and isolation as a barrier for people with dementia living in rural areas. Experts in several countries pointed out that meeting the needs of special groups is a challenge because of their small numbers; for example, there is less knowledge about these groups and they are not a priority for receiving funding and resources. Experts therefore perceived care provision to be necessarily less cost-effective in these cases. The view of some experts was that they had no expertise and no novel ideas, strategies or suggestions concerning special groups. In contrast, some IE experts expressed the view that a truly needs-focused, tailored, coordinated and responsive dementia care system would address many of the ways in which these groups are currently disadvantaged.

*“Well, we have many ideas and have tried many things. But except for caregiver trainings in foreign languages, we do not have accomplished much.” (DE2).*

*“…to try and develop a set of core supports which are at least as viable as you can make them and I think you have to make them consistently… I mean, that has to be your sort of standard…” (IE1).*

### IX. Social Inclusion

Experts in many countries agreed that there seems to be an increasing awareness about dementia.

*“Everyone’s talking about dementia – it’s everywhere.” (UK2).*

*“I get a sense that there’s much more openness to talk about dementia. I think dementia is much more out there, which is great… So I think it’s much more on the agenda. It’s interesting, you see it popping up in all sorts of places that you wouldn’t have before.” (IE7).*

Experts often highlighted the success of different public awareness measures delivered by various organisations. An overriding theme to emerge was that social inclusion should be the ultimate goal of public awareness. This refers to the need to build a society that is open to older people in general and to people with dementia in particular. Knowing someone with dementia or coming into contact with them was viewed by the experts as a powerful means of promoting social inclusion.

*“The aim should not be to have services for people with or without dementia, the aim should be to have services for people in advanced ages…. We have to create normality here.” (DE1).*

*“When people have to engage with the issue themselves in their own families or their own communities, those kind of barriers break down in general and people begin to normalise.” (IE2).*

### X. Raising Awareness

Experts named different approaches to increase public awareness. They referred to the need for education, even as early as in kindergarten or schools.

*“Education is another really important thing. Educating people at a school-going age around dementia, and making them aware at a very young age ...” (IE4).*

They emphasised the importance of continuing to provide the public with accurate information about dementia through awareness and educational campaigns which seek to enhance understanding of dementia and decrease the stigma surrounding it. Experts highlighted the role of mass media (television, movies or books) in conveying these messages. Experts often referred to campaigns and made reference to successful campaigns from other countries that might serve as a model for creating campaigns in their own countries. In that regard, the UK’s “Dementia Friends” campaign was mentioned by experts in many countries. Interestingly, experts from the UK went a step further and recommended aiming for Dementia Friendly communities.

*“For example, when I think of England, there are campaigns such as “Dementia Friends”. It is about informing society and all people about the dementia phenomenon, and it is about training people to deal with people with dementia…. And there is a similar project in Germany that is being funded now.” (DE5).*

*“We’ve got a base level awareness - Dementia Friends is very light touch but it is creating public awareness. How do we then build on that? And that’s where dementia-friendly or dementia-supportive communities come in to play because you are actually asking people to come together, and talk about changes in the way they provide general public services, communities, facilities, retail, outlets and so on in a way that’s more accessible for people with dementia.” (UK3).*

NO experts highlighted a positive shift in attitudes towards dementia during the past years following a large national campaign to raise money and inform about dementia in 2013. IE experts alluded to a citizenship approach of fostering community engagement, i.e., creating a spirit of responsibility of all people in creating awareness and providing support and services around formal dementia care.

*“I’d love to see it going beyond awareness to really creating a much more activated and engaged community. So instead of just leaving it at ‘this is what dementia is’, creating a good message around that that’s positive and so on but to follow it up – ‘and this is what you can do’.” (IE7).*

Involving celebrities was also a suggestion of the experts. Experts from countries that have championship from a high profile figure, such as The Prime Minister of the UK or The Queen of SE, highlighted positive experiences, while experts from countries that lack such prominent championship, such as IE, expressed the wish to be able to involve public figures.

*“And I don’t know in an Irish context, do we need to maybe ask President Higgins to champion this… I mean, there are some celebrities, Pat Kenny, of course, did stuff because his mother had dementia and so on. But, no disregard to him, we need somebody bigger. We need Bono.” (IE6).*

### Xa. Stigma.

Issues of stigma and taboo associated with dementia emerged as a sub-theme of “Raising Awareness”. The theme also includes reference to how stigma can interfere with access and utilization of help and services as well as reference to what measures might reduce stigma. The theme “Stigma” was explicitly addressed by experts in IE, IT, PT and UK and was inductively derived in interviews from NL.

*“There is still a sense of stigma and in particular people have a fear of not being able to cope, and worries about having to live in a care home and this sometimes can put them off asking for help” (UK4).*

*“For ordinary people like a shopkeeper or a Garda [police] to have a tiny bit of training to understand what you can and can’t do, how you can help, when you need to call in expert help would help to reduce stigma and increase understanding…” (IE2).*

## Discussion

Semi-structured qualitative expert interviews were performed to elucidate the perspective of policy and political decision makers in dementia care on three topics of barriers and facilitators identified in previous ActifCare research, i.e., complexity and continuity of care, formal services, and public awareness. A strength of our study is that it pooled important contributions from 38 experts in eight countries regarding a specific, crucial topic in dementia care: access to and utilization of formal home- and community-based services. Notable is the identification of several themes that were common and similar across countries despite the differences in health care systems, culture, traditions, or economic situation. These cross-country similarities enable a more cohesive EU-wide approach to dementia care. Revisiting the common themes across countries reveals keywords such as “Coordination”, “Information”, “Networking”, “Tailored, individualised, multidisciplinary services”, or “Education, mass media and campaigns to raise public awareness”. These keywords are well established among both researchers and practitioners in this area [13–17]. Experts seem to be well aware of barriers and facilitators in their current dementia care systems, and are familiar with current discussions of possible approaches to improve dementia care. Experts may focus on these well-known approaches, because realization and implementation of these approaches into practice is still lacking or not satisfactory. Issues in formal dementia care raised by policy and political decision makers agree with previous policy objectives and recommendations and remain highly salient, a finding that has also been highlighted by Sutcliffe and colleagues [18].

One of the important themes that emerged was the request for a coordinating role. Here, the perspective of experts corresponds well with the perspective of stakeholders in ActifCare focus group interviews [11]. The ActifCare focus group study investigated barriers and facilitators for accessing and using formal care from the perspective of people with dementia, informal carers and healthcare professionals, and one of the major findings was that a key contact person should be established. Thus both in ActifCare expert interviews and in ActifCare stakeholder focus groups, a key coordinating person or institution that is constantly approachable was identified as a potential means of improving pathways to appropriate formal help and services. The need to organize and coordinate dementia care, to link informal and formal care systems, and to manage and shape dementia care pathway are all aspects implied in the request for a coordinating role, and have all been highlighted in past research [19–22].

However, this idea of a coordinating role may be hard to create and implement in practice. In fact, such a coordinating role is widely lacking across the European countries involved in the ActifCare project, except for NO where most of the municipalities have dementia teams or dementia coordinators. Among our experts, there was a notable lack of agreement regarding what structures or systems are needed to realise such a role and regarding skills, competencies and knowledge required to undertake the role. The coordinating role is one of the core consistent findings in this study, being viewed by many experts to be of seminal importance to improve access to and utilization of care, while ideas how to put it into practice are still lacking. Future efforts in research, practice and policy and political decision making should therefore be aimed at defining the role, at examining different approaches to put this role into effect, e.g. different models of case management, and at investigating practical, structural, systemic and economic implications, to see what works best and why.

In relation to the coordinating role, the role of the GP was a matter of debate among the experts. Although the structural system of dementia care in many countries puts the GP in the frontline of navigating and coordinating access to services, GPs may lack the necessary skills and/or resources for such a pivotal role. To solve the issue, either additional supports may be needed by GPs and allied health professionals, or alternatives for the coordinating role may be considered, the latter being the approach taken by some experts in our study.

Regarding information, ideas of the experts included providing online platforms and websites. Problems were simultaneously acknowledged, for example, many older people including people with dementia and their carers may not use a computer or the internet, and many may prefer to receive information personally or interactively with the possibility for questions and counselling. Also, such an approach may be difficult to realize in dementia care. Setting up information platforms and websites requires time and resources to identify what services and supports are actually available, requires efforts to keep it up to date, and requires collaborations across different care institutions and stakeholders, which might not be easy tasks in dementia care systems that lack clear dementia care pathways, lack integrated health and social care, or lack communication between different health professionals and between different sectors. Thus, what at first might appear plausible and fairly obvious, can at most be part of a potential solution.

It is an important finding that experts across countries agreed that formal services should be client- or person-centred, tailored to individual needs, and multidisciplinary. Experts perceive these characteristics to be necessary when influencing access to and utilization of formal dementia services. Experts emphasize that service providers, not people with dementia and their carers, should change to promote access and utilization. This finding can be discussed in relation to the Andersen Model, a theoretical framework of health services use. The most detailed explication of the model posits that health service use is determined by societal factors, health service system factors, and individual factors [23]. The characteristics of services that the experts named in our interviews belong to health service system factors, as opposed to characteristics of people with dementia and carers that the experts did not name and that belong to individual factors. Thus, experts referred to the structural organization of the health services system rather than to individual factors when thinking of innovative ideas how to shape formal dementia services.

Concerning ways to increase public awareness, experts highlighted the necessity of education, mass media and campaigns. These measures perceive responsibility at a structural, political level and can mainly be realized in a top-down fashion. In contrast, IE experts made a point of the concept of community engagement, a measure that perceives responsibility at a community or even individual level and can mainly be realized in a bottom-up fashion. The request for measures to increase public awareness by the experts agrees with well-established research findings [14] and with research priorities that have recently been put forward [24], and thus serves as an example that experts are up-to-date regarding current approaches and discussions of dementia care.

## Limitations and strengths

An important challenge in this study was the conceptualization of the interview topics and the interview guide. Since the expert interviews were conducted in eight different countries with seven different languages, using a jointly developed semi-structured interview guide was considered most appropriate. Using this method had several advantages. First, the topics that were to be discussed with the experts built on barriers and facilitators identified within the same project and were thus based on empirical findings. Second, the topics were found to be important across the ActifCare partner countries and thus represent a basis for the synthesis across countries. Third, an interview guide was used that provided the general topics and a joint questioning route rather than the specific individual questions. We expected that translating general topics and a joint questioning route into the individual languages would result in a more adequate qualitative interview method than translating specific individual questions. The resulting semi-structured interview guide was binding in content yet flexible in form when conducting the individual expert interviews.

To analyse the expert interview data, we applied a stepwise approach involving country-specific inductive qualitative content analysis followed by a cross-national synthesis. Previous research analysing transnational qualitative interview data mainly relied on a consented coding system with joint categories [25, 26]. We chose a modified approach to enable deeper interpretation of latent content in each country. We then synthesized the findings across countries and used discussions by all partners to ensure adequate representation of each country’s findings. The resulting synthesis provides a descriptive summary of similarities in the themes identified in each country; however, it does neither enable explorations of inter-country differences nor descriptions of intra-country differences or agreements among experts. In fact, our analytical strategy provided summarized content of the expert interviews and enabled a description of similarities in the themes across countries, but did not allow a systematic analysis of differences. The synthesis only contains inter-country and intra-country differences if these were apparent during our analytical process; additional differences may have remained undetected. Thus, our synthesis unsystematically refers to some inter-country differences, specifically when themes were not found in interviews with experts from specific countries (e.g. the theme Information was not found in NL and SE) or when certain aspects or examples within an overall theme differed between countries (e.g. the theme Networking/Cooperation/Communication was found across countries while the specifications within the theme differed). For the same reasons, very few intra-country differences were highlighted (e.g. that one single expert from NO perceived dementia services as having received increased funding). The quality criterion of data saturation is often applied in qualitative analysis [27]. In our study, expert interviews were not conducted until data saturation was reached. Instead, the number and the types of experts to be interviewed in each country were predetermined. Most experts were identified at national levels, reflecting that most ActifCare countries either have dementia care systems where influential experts are to be found at national levels or have experts in elderly or dementia care operating on national levels. To identify ideas, strategies and suggestions concerning access to dementia care, sampling adequacy but not solely data saturation may be an appropriate criterion [28], and this has been accomplished in our study by involving a diversity of types of experts and of institutional affiliations of experts in all countries.

## Conclusions

This study elucidates the perspective of policy and political decision makers on core topics related to access to formal home- and community-based dementia care. Several common themes evolved across the eight European countries, indicating qualitative accordance concerning relevant issues in access to this care for people with dementia and their carers. Among others, the experts highlight the need for a coordinating role and the necessity of information to address issues of complexity and continuity of care, demand person-centred, proactive, and multidisciplinary formal services, and refer to education, mass media and campaigns as means to raise public awareness. These ideas, strategies and suggestions show that experts are well acquainted with current discussions among both researchers and practitioners of approaches to improve dementia care. They produced practical measures, such as creating a coordinating role to help people navigate the system, providing websites and databases to inform about available services, or involving celebrities to help raise public awareness about dementia.

Interestingly, experts named innovations that agree with approaches, measures and requests to improve care known from both research and practice, however, experts did not name unknown, never-heard-of innovations. This apparent lack of creativity and innovation may be due in part to our selection of experts, who were required to be influential and to have decisional power within the dementia care system. In our study, it was made clear to the expert that we were interested in innovative ideas, strategies or suggestions concerning formal dementia care grounded in the professional knowledge and experiences of the expert and in the expert’s unique position within the existing dementia care system. Thus, the drive to be realistic and pragmatic while thinking of innovations was paramount. The need for compromise may have hindered the frank expression of overtly new ideas by some of them. Anecdotally, experts from the UK were reported to adamantly refuse to indulge in blue-sky thinking and instead asserted that it was necessary to build on and improve the current situation. This approach is reasonable in our study since we were looking for feasible options to improve access to dementia care. Alternative methods are indicated to investigate truly new, unknown innovations, such as think tanks or open innovation methods, but this was not the focus of the current study.

Our study focuses on dementia care, however, some identified problems may require more structural, systemic changes in the health and social care systems. For example, the necessity of networking, cooperation and communication, the limits and lacks of resources and funding, or the need of a general biopsychosocial perspective may only be overcome by aiming at broader, more profound changes on political, economic, societal or cultural grounds. The findings of our expert interviews have practical implications. Specifically, endorsing a coordinating role that can help with the complexity and continuity of dementia care has the potential to enhance access to and utilization of services by persons with dementia and their informal carers. Future research should identify ways to implement this measure and should investigate its feasibility, effectivity and efficiency. Another core strategy built on insights of our expert interviews relates to raising public awareness regarding dementia. Increasing knowledge and competencies of laypersons, promoting education of the public and fostering attention to dementia issues can directly or indirectly provide support and relief to informal carers.

The experts’ perspective on access to formal dementia will contribute to the core ActifCare aim of developing best-practice strategies to improve the effectiveness and efficiency of access to European dementia care systems and developing recommendations for best practice strategies. Knowledge gained by these expert interviews may be integrated in national decisions to reshape the organisation of dementia care.

## Additional file

Additional file 1: The interview guide used in this study is available as supplementary file with the file name Broda_Suppl_interview guide.pdf. (PDF 53 kb)

## Abbreviations

ActifCare: ACcess to Timely Formal Care
DE: Germany
GP: General practitioner
IE: Ireland
IT: Italy
NL: Netherlands
NO: Norway
PT: Portugal
SE: Sweden
UK: United Kingdom
