# Supplementary material for: Perspectives of policy and political decision makers on access to formal dementia care: expert interviews in eight European countries
Source: BMC Health Serv Res. 2017 Aug 3;17:518. doi: 10.1186/s12913-017-2456-0 (PMC5543593; doi:10.1186/s12913-017-2456-0)
Supplement: Additional file 1: — The interview guide used in this study is available as supplementary file with the file name Broda_Suppl_interview guide.pdf. (PDF 53 kb) [file 12913_2017_2456_MOESM1_ESM.pdf]

ActifCare

WP2 Obj3 Expert interviews

Interview guide

1. Introduce the ActifCare Project, aims and background

The ActifCare Project is a European dementia research project, and is part of the EU Joint Programme - Neurodegenerative Disease Research (JPND). A total of 8 European countries take part in this ambitious project. The project aim is to analyse the pathways to care for people with dementia and their families, in an attempt to better understand barriers and facilitators in access to healthcare. We are especially interested in formal services that are provided for people in the middle stages of dementia living at home, such as day care and home support rather than services such as nursing homes. Our project work included analysing literature as well as conducting qualitative group interviews with people with dementia, their carers, and health care professionals in all 8 countries. In addition, we are currently conducting a survey to repeatedly assess the needs of people with dementia and their carers over one year.

2. Explain what we expect from the expert

We invited you as an expert because you are in a unique position to be influential in dementia care. We expect you possess relevant knowledge and experience on how to influence larger structures and processes and what changes in the overall system are necessary. We invite you to share your professional experience with us, but also to enter into a conversation about innovative ideas, beyond what is already known.

3. Explain the global themes

In the interview, we would like to address selected topics that we identified as common themes in many project countries in focus groups with people with dementia, their carers as well as health care professionals. We would like to discuss with you the topics “A: Complexity and continuity of care”, “B: Formal Services for a heterogeneous group of people with dementia”, and “C: Public awareness”.

A: Complexity and continuity

First, let's talk about the topic **Complexity and continuity** of the dementia care system.

Our interviews with people with dementia and their carers indicated that complexity and lack of continuity of the dementia care system may impede access to and utilisation of necessary services. Complexity refers to e.g. many different types of services and many different ways to access services that may be available and appropriate. Lack of continuity refers to e.g. disjointed and fragmented services, or the absence of a key contact person or institution.

In your expert opinion, what has to happen to enable people with dementia and their carers to better navigate the complexity of the system and to improve the continuity of the system for people with dementia and their carers?

B: Formal Services

B.1: Formal Services for people in the middle stages of dementia living at home

Now we would like to talk about **Services for people in the middle stages of dementia living at home.**

In our interviews with people with dementia and their carers we found that they would like to see a greater consideration of social needs in addition to physical needs. E.g. day care services are often considered as being for people in need of care, in advanced stages of the disease, or are considered to be exclusively oriented to older people, and are therefore not utilized.

In your expert opinion, what should be done to provide this group with appropriate formal services?

B.2: Formal Services for other groups with special needs

In our focus group interviews we also identified special needs for the group of… [refer to your country's "special needs group(s)" as an example].

How can appropriate formal services be created for groups with special needs?

C: Public awareness

Our next topic will be **Public awareness** concerning dementia. This refers to societal attitudes towards dementia and towards people with dementia, which may influence the ways of coping with the disease and the utilisation of help and services.

In your opinion, how does the public perceive dementia and people with dementia?

How can public awareness be effectively targeted?

Suggestions for additional questions, if original answer of the expert is not sufficient *(for all 3 topics):*

- What structures in the dementia care system are necessary to address [topic]?
- How can [topic] be addressed on an overall system level?
- Which measures do you think are promising in influencing [topic]?
- What are barriers and facilitators to address [topic]?
- **Please always ensure focus on people in the middle stages of dementia living at home,** e.g.: How is that different for people with dementia living at home? What structures/which measures are necessary for people in the middle stages of dementia?

Last question

To conclude we ask you to share your vision of the ideal dementia care system with us. Imagine you could redesign the existing system. How would you shape the dementia care system to optimize access to care?

Are there any aspects you feel are important but we haven't talk about yet?
